# Morphologic identification of clinically encountered moulds using a residual neural network

**DOI:** 10.3389/fmicb.2022.1021236

**Published:** 2022-10-14

**Authors:** Ran Jing, Xiang-Long Yin, Xiu-Li Xie, He-Qing Lian, Jin Li, Ge Zhang, Wen-Hang Yang, Tian-Shu Sun, Ying-Chun Xu

**Affiliations:** ^1^Department of Laboratory Medicine, State Key Laboratory of Complex Severe and Rare Diseases, Peking Union Medical College Hospital, Chinese Academy of Medical Science and Peking Union Medical College, Beijing, China; ^2^Graduate School, Chinese Academy of Medical Science and Peking Union Medical College, Beijing, China; ^3^Beijing Key Laboratory for Mechanisms Research and Precision Diagnosis of Invasive Fungal Diseases, Beijing, China; ^4^Beijing Hao Chen Xing Yue Technology Co., Ltd., Beijing, China; ^5^Beijing Xiaoying Technology Co., Ltd., Beijing, China; ^6^Medical Research Center, State Key Laboratory of Complex Severe and Rare Diseases, Peking Union Medical College Hospital, Chinese Academy of Medical Sciences and Peking Union Medical College, Beijing, China

**Keywords:** ResNet-50, XMVision Fungus AI, clinical moulds, rapid identification, morphology

## Abstract

The use of morphology to diagnose invasive mould infections in China still faces substantial challenges, which often leads to delayed diagnosis or misdiagnosis. We developed a model called XMVision Fungus AI to identify mould infections by training, testing, and evaluating a ResNet-50 model. Our research achieved the rapid identification of nine common clinical moulds: *Aspergillus fumigatus* complex, *Aspergillus flavus* complex, *Aspergillus niger* complex, *Aspergillus terreus* complex, *Aspergillus nidulans*, *Aspergillus sydowii/Aspergillus versicolor*, *Syncephalastrum racemosum*, *Fusarium* spp., and *Penicillium* spp. In our study, the adaptive image contrast enhancement enabling XMVision Fungus AI as a promising module by effectively improve the identification performance. The overall identification accuracy of XMVision Fungus AI was up to 93.00% (279/300), which was higher than that of human readers. XMVision Fungus AI shows intrinsic advantages in the identification of clinical moulds and can be applied to improve human identification efficiency through training. Moreover, it has great potential for clinical application because of its convenient operation and lower cost. This system will be suitable for primary hospitals in China and developing countries.

## Introduction

Invasive fungal diseases are life-threatening infections, and main fungal pathogens that cause invasive infections have been getting more and more attention in the last decades. A study from China reported that candidiasis was the most common invasive fungal disease, with a morbidity of 45.3% (Gong et al., 2020). In recent years, studies have shown that invasive infections caused by moulds are gradually increasing, mainly threatening patients with immune systems compromised by malignant tumours, neutropenia, organ transplants, diabetes, AIDS, and other conditions (Li et al., 2020). Invasive mould infections can occur deep tissues, and even in the central nervous system (Jing et al., 2022); and skin lesions may occur as a consequence of the systemic dissemination of invasive moulds, or after direct inoculation in the skin (Tessari et al., 2014). In particular, aspergillosis, which is caused by *Aspergillus* spp., is the most common mould infection in humans, contributing >85% of invasive mould infections and leading to a high mortality of >50% (Mccoy et al., 2009). An increase in the incidence of aspergillosis from 11.1% in 1998 to 28.8% in 2018 has been observed (Gong et al., 2020).

Morphologic identification has always played a dominant role in mould identification. Because of the diversity of morphologic characteristics of the colonies, conidia, conidiophores, and other structures in different mould taxa, the morphologic identification is a difficult job for novices, who lack experience and expert knowledge of mycology. However, most of primary hospitals in China lack professional microbiologists and fungal diagnostic consultation resources, which often leads to delayed diagnosis or misdiagnosis of invasive mould infections. In addition, traditional morphologic identification is time-consuming and inefficient enough to handle the increasing number of specimens in most general hospitals in China. In recent years, artificial intelligence (AI) has greatly advanced the development of laboratory medicine, including the identification of blood cells, cancer cells, body fluids components, and the identification of bacteria/fungi in digital images (Esteva et al., 2017; Ho et al., 2019; Anita and Yadav, 2021; Bhinder et al., 2021; Ma et al., 2021; Sh et al., 2021; Wang et al., 2021). Given the challenges of morphologically based diagnosis of invasive mould infections, it is necessary to develop a rapid and accurate mould identification technique *in vitro*. In our study, we developed a model called XMVision Fungus AI for use in mould identification. This model is based on the ResNet-50 architecture, a type of deep convolutional neural network, and was developed through training, testing, and evaluation. Our research has performed the rapid and accurate identification of nine mould taxa of clinically moulds, *Aspergillus fumigatus* complex, *Aspergillus flavus* complex, *Aspergillus niger* complex, *Aspergillus terreus* complex, *Aspergillus nidulans*, *Aspergillus sydowii/Aspergillus versicolor*, *Syncephalastrum racemosum*, *Fusarium* spp., and *Penicillium* spp., and the overall accuracy rate reached 93.00%.

## Materials and methods

### Data collection

A total of 398 strains with different sample ID numbers covering the 39 mould taxa as an initial fungal resource (Supplementary Table S1) identified by the China Hospital Invasive Fungal Surveillance Net-North China Program from 2017 to 2018 were cultured on Potato Dextrose Agar (Thermo Fisher Scientific, Waltham, MA, United States) and Sabouraud Dextrose Agar (Thermo Fisher Scientific, Waltham, MA, United States) and incubated for 3–7 days at 28°C. All strains were initially identified using conventional morphologic methods combined with matrix-assisted laser desorption ionization time-of-flight mass spectrometry (MALDI-TOF MS).

**Figure 1:**
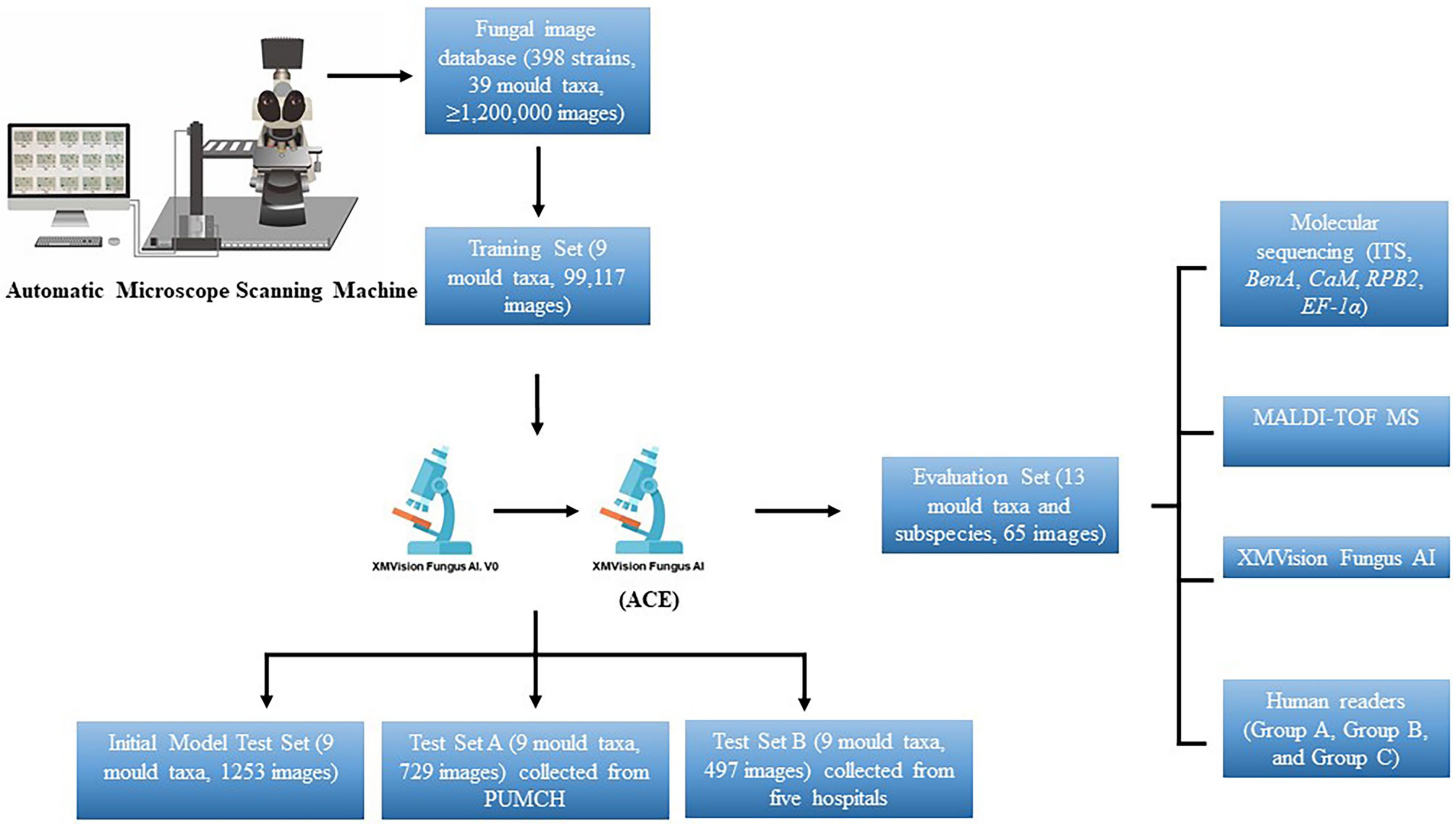
Overall process to create XMVision Fungus AI from training, testing, to evaluation. No matter what brand of microscope or camera is used, but a trinocular light microscope with sleeve size in 0.5x, and a camera mounted on the microscope with a target size in 1/1.8 inch, are required to both automatically or manually capture images. At the same time, a visual tool software (e.g., MGview system) matching the camera needs to be downloaded on the computer, to enlarge the dynamic microscopic fields and capture the target areas as screenshots. ACE, adaptive contrast enhancement; MALDI-TOF MS, matrix-assisted laser desorption ionization time-of-flight mass spectrometry; PUMCH, Peking Union Medical College Hospital; ITS, internal transcribed spacer regions of ribosomal DNA; *BenA*, β-tubulin; *CaM*, calmodulin; *RPB1*, RNA polymerase I subunit; *RPB2*, RNA polymerase II subunit; *EF-1α*, elongation factor alpha subunit.

The overall process used in this study to create the XMVision Fungus AI consists of the training, testing, and evaluation of a ResNet-50 model, as summarized in Figure 1. Prior to ResNet-50 model training, we cultured the selected strains under the conditions described above, and specimen slides were prepared using lactophenol cotton blue staining and the cellotape flag method according to direct microscopic examination procedures. The prepared slides were first observed under a microscope to ensure the quality and typical appearance of each slide. An automatic microscope scanning machine, as shown in Figure 1, was used to scan the prepared slides under a 400x microscope, obtaining 3,000 to 5,000 images per slide. Thus, over 1,200,000 images were scanned and stored in the fungal image database. Because of the limited number of strains of each species, clinically encountered moulds with more than 10 strains in each species were selected for training. In addition, a custom image-processing program was developed to screen images and eliminate invalid images with a large number of bubbles, an atypical microscopic appearance, only hypha, or even an empty field. As a result, 90% of the total images were automatically eliminated, leaving approximately 120,000 images for manual eliminating. Of these, 99,117 images (about 10,000 images per species) were used to create the training dataset.

The initial XMVision Fungus AI after ResNet-50 model training was tested on the initial model test set, which consists of 1,253 images (out of the training set) from the initial fungal image database. To evaluate the accuracy of our model, test set A, which consists of 729 images was manually acquired from the Peking Union Medical College Hospital; an additional test set B, which consists of 497 images, was manually collected from five hospitals. Of note, these strains covering the nine mould taxa used to test set A and test set B were independently collected from PUMCH and other five hospitals, separately. Based on the results of the two test sets obtained by the initial XMVision Fungus AI, we analysed the performance and optimized the model to generate the final version of XMVision Fungus AI. The mould identification accuracy of XMVision Fungus AI was evaluated again on the two test sets.

In short, a training set of 99,117 images, initial model test set of 1,253 images, and test set A of 729 images were created to evaluate the accuracy of mould identification in the same setting in Peking Union Medical College Hospital. Moreover, a test set B of 497 images was created to evaluate its performance in different settings.

### Development of the ResNet-50 model for rapid morphologic identification

Many recent studies have demonstrated that the ResNet model is a state-of-the-art image identification model (Szegedy et al., 2016; Kensert et al., 2018; Wieslander et al., 2018; Ilhan et al., 2021). The residual structure of ResNet-50 ensures that the network effectively avoids the gradient vanishing/exploding and network degradation problems by fully learning the deep features of an image. Moreover, the 50-layer network depth keeps the network size manageable (He et al., 2016).

**Figure 2:**
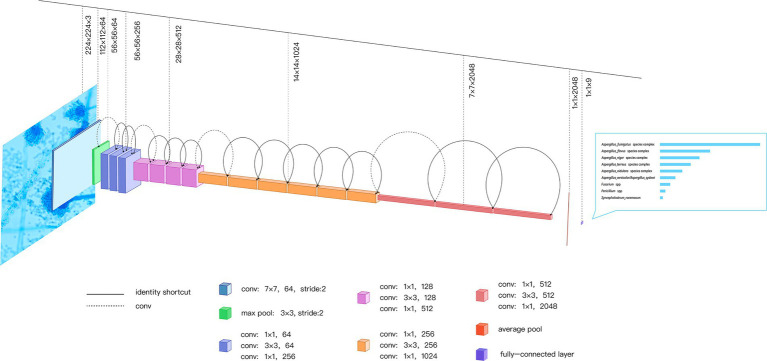
ResNet-50 Network Built-in Blocks. The ResNet-50 consists of 49 convolution layers and a maximum pooling layer; the input image passes through ResNet-50 to get the final identification result. As an example, a microscopic image of one *Aspergillus fumigatus* isolate is passed through the network to get the confidence scores of identification. conv, convolution.

As shown in Figure 2, the input image passes through a convolution layer with a convolutional size of 7 × 7 and a stride of 2 to obtain the downsampled feature. The feature then passes through a pooling layer with a size of 3 × 3 to obtain a new downsampling feature. The feature passes through the first residual block, which contains a 1 × 1 convolution, 3 × 3 convolution, and 1 × 1 convolution, to obtain the initial output of the residual block. The output is summed with the input of the residual block element-wise, and the final output of the residual block is obtained. The output is fed to the next residual block and the input of the next residual block is obtained using the above principle. After 16 residual blocks, the feature of the final residual block output is obtained. This feature is fed to an average pooling layer of size 7 × 7 to obtain the feature map that is ultimately used for identification. The feature is activated by a softmax function to obtain the final network prediction result.

### Image preprocessing for the ResNet-50 model

Because there are significant differences in the background colour and brightness of images collected using different acquisition settings, to improve the robustness of image identification, the chromaticity of the fungal images is normalized before identification. Adaptive contrast enhancement (ACE) is a method for normalizing the images that consists of the following three steps:

Step 1: Normalize the image colour to remove colour deviation caused by different acquisition settings.

The aim is to make brightness of each RGB colour channel equal, that is, ∑(*Y*_r_) = ∑(*Y*_g_) = ∑(*Y*_b_). The weight *W*_i_ is calculated as follows:

Wi = ∑(Xr + Xg+ Xb) / (3*∑(Xi))

to obtain the output *Y*_i_ as

Yi = Xi*Wi,

where *i* ∈ r, g, b (red, green, and blue channels).

Step 2: Normalize the image brightness to solve the problem of inconsistent camera exposure during manual acquisition.

The aim is to change the brightness such that the maximum brightness of image pixels is equal to the brightness limit value, so that Max(Y) = 1, Y ∈ [0,1]. The weight W is calculated as follows:

W = 1/max (X), X ∈ [0,1]

to obtain output *Y* as

Y = X*W.

Step 3: Adaptive contrast equalization between different regions of the image. Bright spots appearing in the centre of some microscope slide images are removed because they can break the translational invariance of the convolutional neural network. This step is accomplished by calling the contrast limited adaptive histogram equalization function of OpenCV.

**Figure 3:**
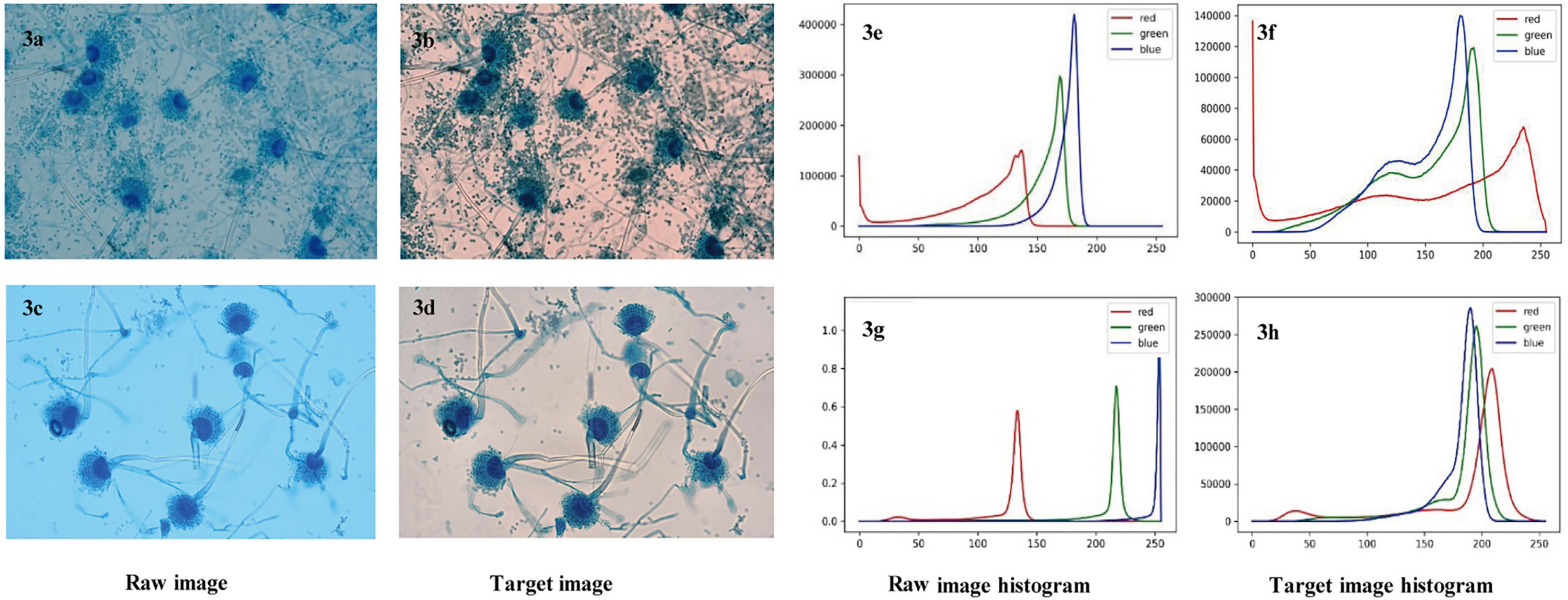
**(A–H)** Characteristic comparisons of raw images and ACE processed target images. [A,C] and [B,D] are two examples of raw and target images of *Aspergillus fumigatus*, respectively, with histograms of [E-H] corresponding to the images of [A-D], respectively. RGB curves represent three color channels which are one of the most widely used color systems. ACE, adaptive contrast enhancement; RGB, red, green, and blue.

From the two original images and two target images shown in Figures 3A–D, it can be seen that the texture of the image after ACE processing is clearer than that of the raw image. Moreover, and the histograms of the raw images have obvious differences in the distribution of the RGB (red, green, blue) component peaks (Figures 3E,G). The histograms corresponding to the two target images after ACE processing are close to the position of the RGB component peaks (Figures 3F,H). ACE processing normalizes the image colour distribution to the same scale, eliminating the image differences caused by the acquisition device and batch. These images are an intermediate step in the identification process, and the purpose of the ACE algorithm is to normalize the background colour and brightness to reduce overfitting, not to restore it to the natural state observed by human eyes.

### Accuracy assessment of XMVision Fungus AI on the morphologic identification of clinical common moulds

An evaluation set consisting of 65 images including images of all 13 strains (out of the initial fungal resource; Supplementary Table S2) of the nine mould taxa and their subspecies that were successfully identified by XMVision Fungus AI was constructed. The aim was to assess the accuracy of mould identification of different methods, including molecular sequencing methods, MALDI-TOF MS, XMVision Fungus AI, and human readers. Molecular sequencing is recognized as the golden standard of species identification. Nucleotide sequencing of the internal transcribed spacer (ITS) regions of ribosomal DNA, the β-tubulin (*BenA*), calmodulin (*CaM*), RNA polymerase I subunit (*RPB1*), RNA polymerase II subunit (*RPB2*), and elongation factor alpha subunit (*EF-1α*) genes of the 13 strains was performed by Beijing Ruibio BioTech Co., Ltd. (Beijing, China), then were homologically aligned with reliable sequences in the GenBank database.^1^ The VITEK^®^ MS V3.2 system (bioMérieux, Marcy-l’Étoile, France) was used in this experiment to obtain the MALDI-TOF MS results for mould identification.

In our study, nine human readers with different levels of mould morphologic identification experience were invited to participate in the testing and divided into three groups (group A, group B, and group C). Group A consisted of three mould morphologists, group B consisted of three technicians in the field of microbiology who had received morphologic training at Peking Union Medical College Hospital, and group C consisted of another three technicians in the field of microbiology who lacked of morphologic training. They performed direct image identification testing made up of the 65 images of the moulds. The identification results from the human readers were then anonymously statistically analysed, and finally compared with the results from XMVision Fungus AI. Here, a Top-1 confidence above 90% was defined as a ‘credible’ result of mould identification, whereas 80–90% and below 80% were defined as ‘somewhat-credible’ and ‘not-credible’ mould identification, respectively.

### Data analysis methods

GraphPad Prism software was used to generate graphs as well as for statistical analyses. Fisher’s exact test and the Chi-square test were used in one-and two-sided comparisons, respectively. Significant changes were defined as *p <* 0.05.

## Results

### Development of the XMVision Fungus AI model

After the images were fed into the convolutional neural network, the computer automatically extracted features from large samples for end-to-end autonomous learning without structured labels. Therefore, the image feature extraction included the background colour and brightness in addition to the microscopic morphologies of fungi, such as the structure of conidiophore and the size of conidia.

According to the training procedure described in Materials and Methods, a total of nine taxa of clinically moulds were selected and successfully identified (*Aspergillus fumigatus* complex, *Aspergillus flavus* complex, *Aspergillus niger* complex, *Aspergillus terreus* complex, *Aspergillus nidulans* complex, *Aspergillus sydowii*/*Aspergillus versicolor*, *Syncephalastrum racemosum*, *Fusarium* spp., and *Penicillium* spp.).

A higher level of Top-1 confidence (above 90%) could be obtained as long as the initial colony culture conditions, standard operations of slide preparation, and matching settings were similar to those of the initial XMVision Fungus AI training conditions. We tried to input some images of other untrained mould taxa, such as *Mucor* spp. and *Aspergillus clavatus*, the identification results were within a very low confidence of Top-1 less than 40%, as ‘not-credible’ results.

### Effect of raw and ace processed images on XMVision Fungus AI performance

**Figure 4:**
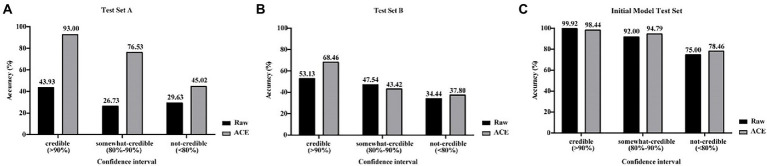
**(A–C)** Comparison of raw and ACE processed images for XMVision Fungus AI performance. Raw images of from initial model test set and manually collected test sets (test set A and test set B) and corresponding ACE processed images were used to assess the mould identification performance of XMVision Fungus AI, with a TOP-1 confidence above 90% defined as a ‘credible’ result of mould identification, whereas 80–90% and below 80% defined as ‘somewhat-credible’ and ‘not-credible’ mould identification, respectively. ACE, adaptive contrast enhancement.

Mould identification performance of XMVision Fungus AI was evaluated using raw images of from the initial model test set and manually collected test sets (test sets A and B; Figures 4A–C). As a whole, the accuracy of raw image identification on the initial model test set was higher than that on test set A, with a credible range of 99.92% vs. 43.93%, a certain confidence range of 92.00% vs. 26.73% and an untrusted range of 75.00% vs. 29.63%, respectively.

By analysing the differences in the images of test set A and those of the initial model test set, such as the brightness or colour of the background, we found that ACE reduced these differences between the manually collected and initial model test sets. After processing the raw input images using ACE, the mould identification accuracy of test set A was significantly increased (from 43.93 to 93.00%), and mould identification accuracy of test set B were slightly increased (from 53.13 to 68.46%) within each credible range. In addition, the accuracy of test set A was a substantially increased (from 26.73 to 76.53%), and the accuracy of test set B was slightly reduced (from 47.54 to 43.42%), within the certain confidence range. Finally, test sets A and B both increased slightly (from 29.63 to 45.02% and 34.44 to 37.80%), within the untrusted range (Figures 4A,B). However, the accuracy of the initial model test set was slightly reduced, within the credible range (from 99.92 to 98.44%), and was slight increased within the certain confidence range (from 92.00 to 94.79%) and within untrusted range (from 75.00 to 78.46%; Figure 4C). These results demonstrated that processing the input images using ACE increased the mould identification performance of XMVision Fungus AI on the manually collected images (*p* < 0.0001), especially for test set A (*p* < 0.0001). This was a reasonable result that revealed that the raw image identification on the initial model test set had higher accuracy but relied too much on the same image acquisition settings as the training set (such as the same batch, incubation time, and background colour). ACE processing improved the identification performance of XMVision Fungus AI for images acquired from different settings. In addition, the higher identification accuracy of raw images of initial model test set (99.92% within the credible range) could not be interpreted, possibly as a result of the overfitting phenomenon in image identification. It is difficult to avoid the overfitting phenomenon, which may lead to a higher accuracy in identifying the training set but a poorer accuracy in identifying sets of independently evaluated images (Ma et al., 2021).

### Performance of XMVision Fungus AI on images from different sets

**Figure 5:**
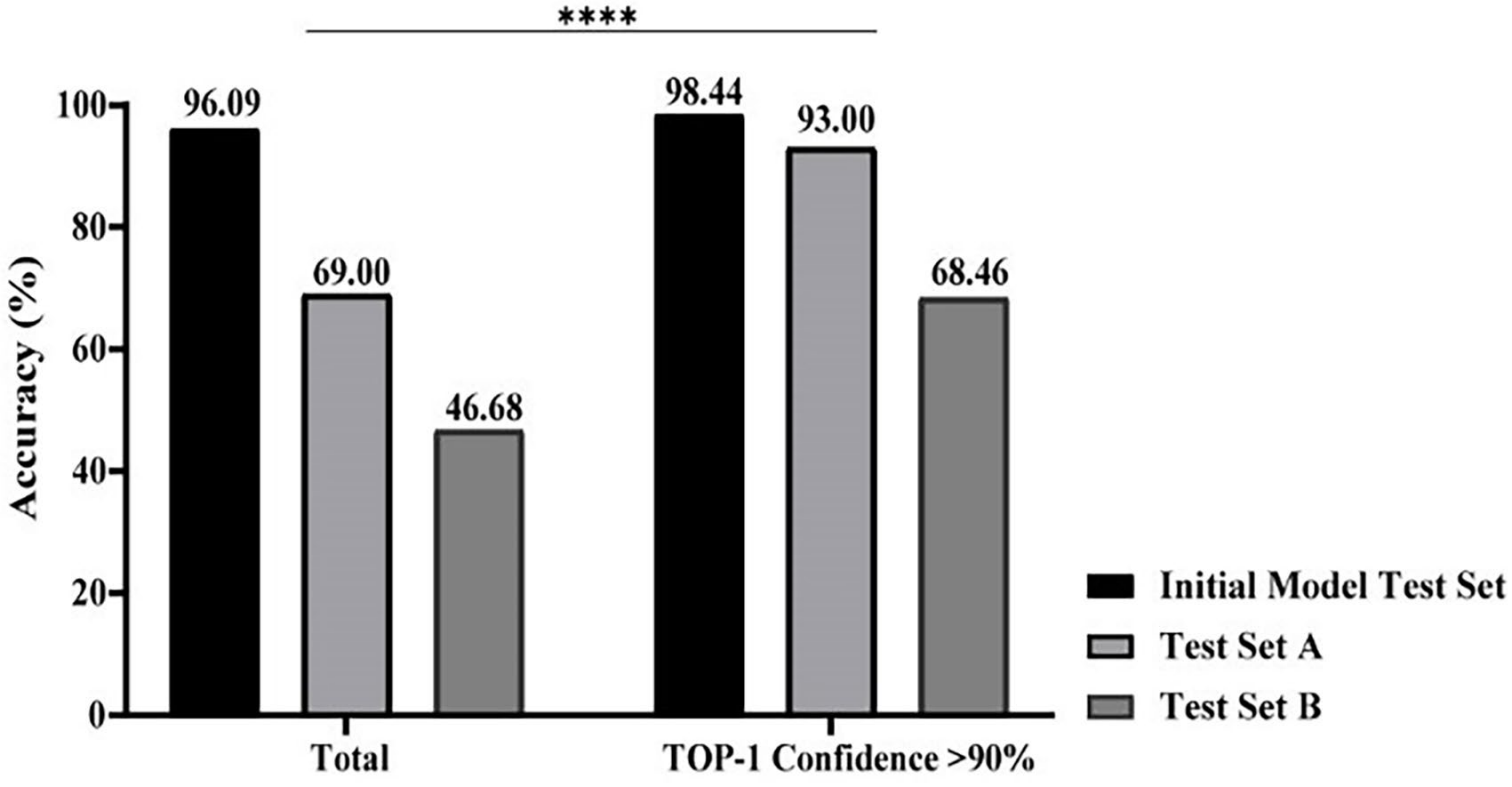
Performance comparison of XMVision Fungus AI on images from different sets. A total of accuracies of mould identification were compared with or without a constraint of a TOP-1 confidence above 90% (Chi-square, ****, *p* < 0.0001).

Based on ACE processed images, when the Top-1 confidence value was above 90%, the overall accuracies of mould identification of test set A (*n* = 729), test set B (*n* = 497), and the initial model test set (*n* = 1,253) increased to 93.00% (279/300), 68.46% (89/130), and 98.44% (1,011/1027), respectively; when compared with the lower accuracies of 69.00% (503/729), 46.68% (232/497), and 96.09% (1,204/1253) obtained in each set without the confidence constraint (Figure 5; *p* < 0.0001). Therefore, it was necessary to add threshold of a confidence above 90% (*p* < 0.0001) as a constraint before evaluating the identification accuracies of each taxon from different sets using XMVision Fungus AI (Table 1). The accuracies of training set and initial model test set of arbitrary taxon ranged from 86.71 to 99.95% and from 92.73 to 100%, respectively, showing the high ability of XMVision Fungus AI to distinguish taxon from the same acquisition setting. Its overall accuracy of test set A is relatively satisfactory (93.00%; Figure 5), especially for identification accuracies over 95.00%: *Aspergillus niger* complex (100%), *Aspergillus nidulans* complex (97.87%), and *Fusarium* spp. (96.72%). The exceptions were *Aspergillus versicolor/ Aspergillus sydowii* species, as only three images were screened from test set A. However, the identification accuracies of test set A for the *Aspergillus flavus* complex and *Penicillium* spp. were relatively low, at 70.97 and 87.88%. It is worth noting that, the overall accuracy of test set B was low, at 68.46% (Figure 5), which includes images from different acquisition settings. After excluding mould taxa with fewer than 10 images in test set B, identification accuracies of less than 80% were found for *Penicillium* spp., *Aspergillus fumigatus* complex, and *Aspergillus flavus* complex (42.86, 57.58, and 56.52%, respectively).

**Table 1:** Evaluation of identification accuracies of each mould taxon from different sets by XMVision Fungus AI.

Mould taxa	Training set (n)	Training set (%)	Initial model test set (n)	Initial model test set (n)	Test set A (n)	Test set A (%)	Test set B (n)	Test set B (%)
*A. fumigatus species complex*	10,150	94.62	73	100	29	93.10	33	57.58
*A. flavus species complex*	7,437	86.71	59	94.92	31	70.97	23	56.52
*A. niger species complex*	9,771	99.60	183	99.45	41	100	27	88.89
*A. terreus species complex*	8,077	99.92	99	100	38	94.74	11	100
*A. nidulans species complex*	10,251	99.92	102	100	47	97.87	3	66.67
*A. versicolor/Aspergillus sydowi*	12,158	99.95	165	92.73	3	100	3	33.33
*Fusarium* spp.	9,421	99.84	95	100	61	96.72	14	85.71
*Penicillium* spp.	12,556	99.94	158	100	33	87.88	14	42.86
*S. racemosum*	9,597	99.91	93	100	17	94.12	2	50
Total	89,418	98.18	1,027	98.44	300	93.00	130	68.46

### Performance comparison of XMVision Fungus AI, human readers, and MALDI-TOF MS, based on molecular sequencing identification

**Figure 6:**
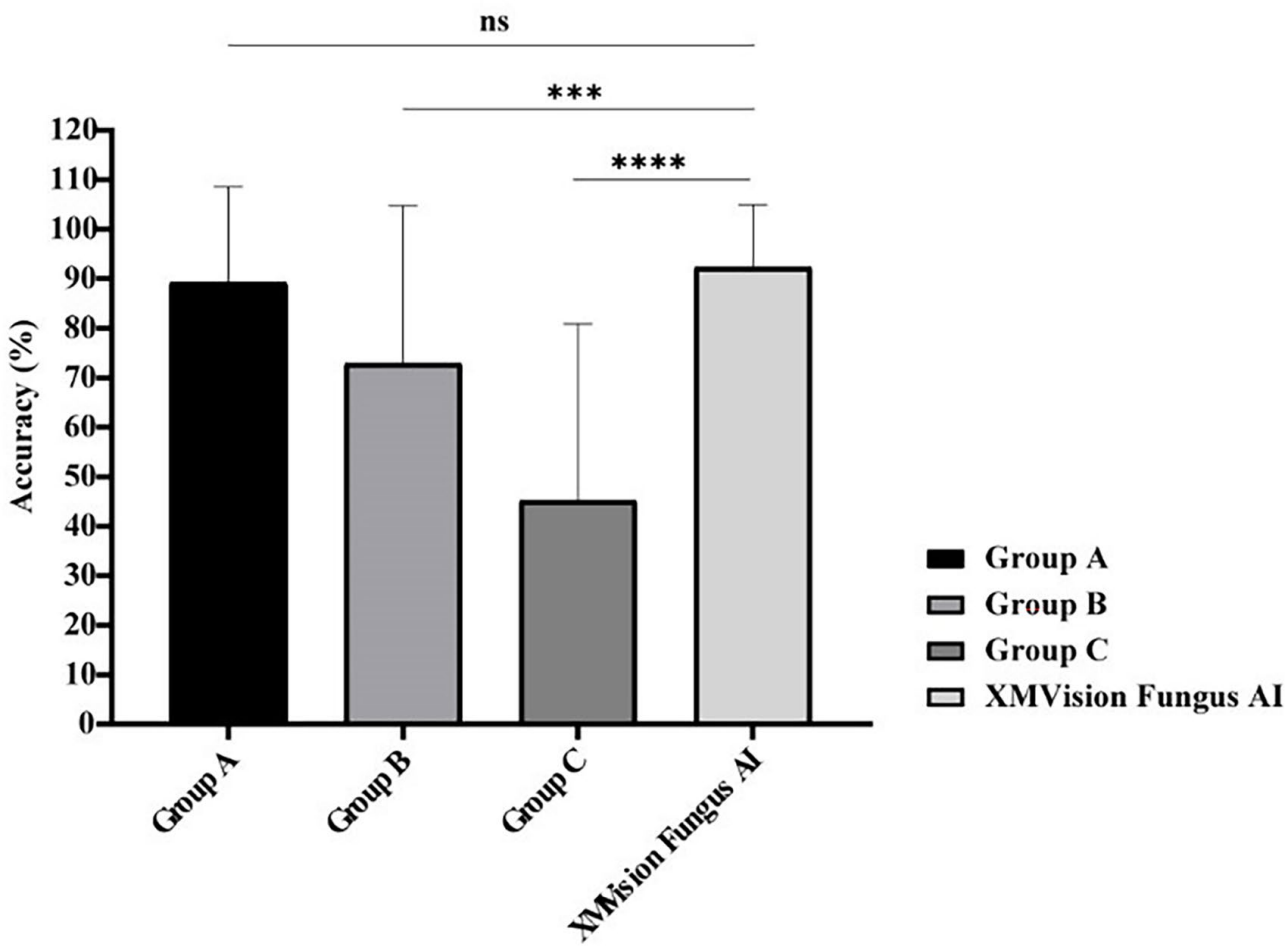
Performance comparison of mould identification between XMVision Fungus AI and human readers with different levels. Group A consisted of three mould morphologists, group B consisted of three technicians in the field of microbiology who had received morphologic training at Peking Union Medical College Hospital, and group C consisted of another three technicians in the field of microbiology who lacked of morphologic training. PUMCH, Peking Union Medical College Hospital. (Mean + standard deviation from three independent tests, two-way ANOVA, ns, *p* = 0.3369; ***, *p* = 0.0003; ****, *p* < 0.0001).

The identification results of *Aspergillus* species at the complex level and other moulds including the *Penicillium* and *Fusarium* species at the genus level were regarded as correct in these results. Comparison between the mould identification results from the human readers and XMVision Fungus AI found that XMVision Fungus AI obtained the overall highest accuracy of 92.31%. Groups B and C, who with lower accuracies of 72.82 and 45.13% (*p* = 0.0003 and *p* < 0.0001), respectively (Figure 6). There was no significant difference in the mould identification performance of XMVision Fungus AI and Group A (accuracy of 89.23%; *p* = 0.3369; Figure 6). Focusing on the mould identification result of the human readers (Figure 6), it indicated that the ability of morphologic mould identification of human readers could be improved by training.

**Figure 7:**
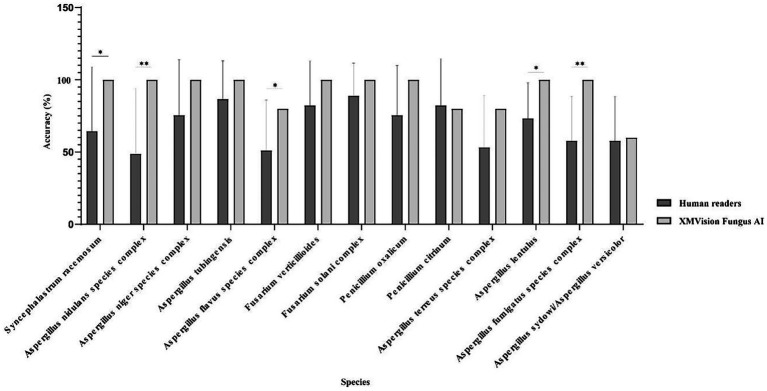
Performance comparison of each taxon identification between XMVision Fungus AI and human readers (Paired, two-tailed t test, *, *p* = 0.0433, 0.0375, 0.0114; **, *p* = 0.0091, 0.0033).

The XMVision Fungus AI identification performance for the taxa *Syncephalastrum racemosum*, *Aspergillus nidulans* complex, *Aspergillus flavus* complex, *Aspergillus lentulus*, and *Aspergillus fumigatus* complex was significantly better than that of human readers, with accuracies of 100% vs. 64.44% (*p* = 0.0433), 100% vs. 48.89% (*p* = 0.0091), 80% vs. 51.11% (*p* = 0.0375), 100% vs. 73.33% (*p* = 0.0114), and 100% vs. 57.78% (*p* = 0.0033), respectively (Figure 7). Only human readers had slightly higher identification ability for *Penicillium citrinum* than XMVision Fungus AI (82.22% vs. 80.00%, *p* = 0.8417; Figure 7). In addition, neither XMVision Fungus AI nor the human readers were able to accurately identify *Aspergillus sydowii*/*Aspergillus versicolor*, with accuracies of only 60.00 and 57.78%, respectively (*p* = 0.8337; Figure 7).

The identification results of MALDI-TOF MS and XMVision Fungus AI were compared based on the results of the molecular sequencing method (Table 2). In the evaluation set, MALDI-TOF MS was able to accurately identify more moulds at the species/subspecies level, including *Aspergillus nidulans*, *Penicillium citrinum*, *Aspergillus fumigatus*, and *Aspergillus lentulus*, with high confidence scores close to 100%. By contrast, XMVision Fungus AI only provided accurate taxa identification for *Syncephalastrum racemosum* and *Aspergillus nidulans*. *Fusarium verticillioides* was not distinguished from *Fusarium proliferatum*, but *Fusarium solani* could be accurately identified by MALDI-TOF MS. These three species were correctly identified as *Fusarium* spp. by XMVision Fungus AI. Similarly, *Penicillium citrinum* and *Penicillium oxalicum* could be correctly identified as *Penicillium* spp. by XMVision Fungus AI, with accuracies of 80 and 100%, respectively, while MALDI-TOF MS performed exact identification ability of *Penicillium citrinum*. Other *Aspergillus* spp., including *Aspergillus niger*, *Aspergillus tubingensis*, *Aspergillus flavus*, and *Aspergillus terreus*, were only identified at the *Aspergillus* complex level by both methods. In addition, *Aspergillus lentulus* was not distinguished from *Aspergillus fumigatus* but was identified at *Aspergillus fumigatus* complex by XMVision Fungus AI. Similar to previous studies (Libert et al., 2017), our results show that *Aspergillus sydowii* and *Aspergillus versicolor* were difficult to distinguish both morphologically and genetically. Due to the absence of database entries for *Syncephalastrum racemosum* and *Penicillium oxalicum* in the VITEK^®^ MS system, no identification results were obtained by MALDI-TOF MS for these species.

**Table 2:** Comparison of mould identification performance by XMVision Fungus AI, and MALDI-TOF MS based on molecular sequencing identification.

Strain ID	Standard method		Compared methods			
	Tagrget genes	Molecular sequencing	MALDI-TOF MS	Confidence score (%)	XMVision Fungus AI	Accuracy (%)
No. 1	*ITS, BenA, CaM*	*S. racemosum*	no result	NA	*S. racemosum*	100
No. 2	*ITS, BenA, CaM*	*A. nidulans*	*A. nidulans*	99.7	*A. nidulans*	100
No. 3	*ITS, BenA, CaM*	*A. niger*	*A. niger* complex	99.9	*A. niger* complex	100
No. 4	*ITS, BenA, CaM*	*A. tubingensis*	*A. niger* complex	99.9	*A. niger* complex	100
No. 5	*ITS, BenA, CaM*	*A. flavus*	*A. flavus/oryzae*	99.9	*A. flavus complex*	80
No. 6	*ITS, EF-1α*	*Fusarium verticillioides*	*Fusarium verticillioides/ Fusarium proliferatum*	99.9	*Fusarium* spp.	100
No. 7	*ITS, EF-1α*	*Fusarium solani*	*Fusarium solani* complex	99.9	*Fusarium* spp.	100
No. 8	*ITS, RPB1, RPB2*	*Penicillium oxalicum*	no result	NA	*Penicillium* spp.	100
No. 9	*ITS, RPB1, RPB2*	*Penicillium citrinum*	*Penicillium citrinum*	99.9	*Penicillium* spp.	80
No. 10	*ITS, BenA, CaM*	*A. terreus*	*A. terreus* complex	99.9	*A. terreus* complex	80
No. 11	*ITS, BenA, CaM*	*A. lentulus*	*A. lentulus*	99.9	*A. fumigatus* complex	100
No. 12	*ITS, BenA, CaM*	*A. fumigatus*	*A. fumigatus*	99.9	*A. fumigatus* complex	100
No. 13	*ITS, BenA, CaM*	*A. sydowi/Aspergillus versicolor*	*A. sydowi/Aspergillus versicolor*	99.9	*A. sydowi/Aspergillus versicolor*	60

MALDI-TOF MS, matrix-assisted laser desorption ionization time-of-flight mass spectrometry; ITS, internal transcribed spacer regions of ribosomal DNA; *BenA*, β-tubulin; *CaM*, calmodulin; *RPB1*, RNA polymerase I subunit; *RPB2*, RNA polymerase II subunit; *EF-1α*, elongation factor alpha subunit; NA, not available.

### Commonly misidentified mould taxa by XMVision Fungus AI and human readers

**Figure 8:**
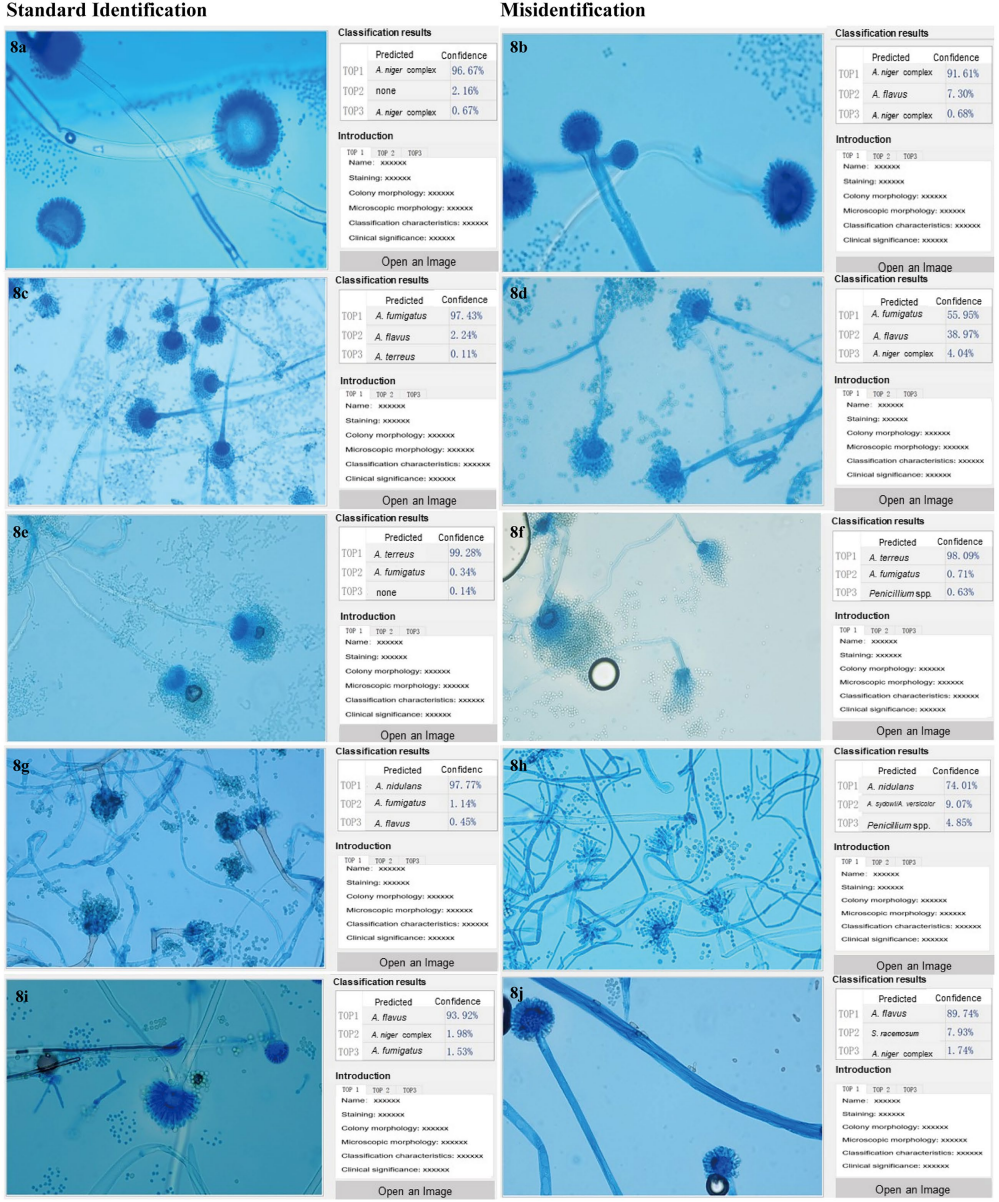
**(A–J)** Commonly misidentified mould taxa by XMVision Fungus AI and human readers. The mould taxa frequently misidentified with each other are listed in Figure 8, including *Aspergillus flavus* complex and *Aspergillus niger* complex, *Aspergillus fumigatus* complex and *Aspergillus flavus* complex, *Aspergillus fumigatus* complex and *Aspergillus terreus* complex, *Aspergillus sydowi/Aspergillus versicolor* and *Aspergillus nidulans* complex, and *Aspergillus flavus* complex and *Syncephalastrum racemosum*. The images of misidentified mould taxa with corresponding standard mould taxa are listed on the right and left half of Figure 8, respectively, all the images captured under a 400x microscope. On the left side of each screenshot box shows the uploaded microscopic images of each mould taxon; In the upper right corner of each screenshot box shows the results of mould taxa identification expressed as three levels of confidence as Top1, Top2 and Top3, and Top1 is recognized as the most credible result, followed by Top2 and Top3 as the second and third possible identification results, respectively. In the lower right corner of each screenshot box shows some reference information for the mould taxa including the staining method, magnification times, colony and microscopic appearances, differential diagnosis, and clinical importance. Notably, due to a large number of words in the reference information, and all the description is in Chinese, so the detailed contents are omitted in the figures.

The image identification results of human readers and XMVision Fungus AI on the initial model test set and manually collected test sets are shown in Supplementary Tables S3A–C, respectively. There were several commonly misidentified mould taxa in the different sets, as summarized in Figures 8A–J.

First, *Aspergillus flavus* complex was easily misidentified as *Aspergillus niger* complex by both XMVision Fungus AI (*n* = 11) and human readers (*n* = 4). It was observed generally that the morphology of *Aspergillus flavus* complex (Figure 8B) resembled the juvenile stage of *Aspergillus niger* complex, as shown in Figure 8A, because both have hemispherical heads. However, the conidiophore of *Aspergillus flavus* complex appeared as roughened stipes and globose vesicles with radiate or columnar spore production, as well as present metulae, with uniseriate or biseriate phialides radiating directly to the entire surface of the vesicle (Figure 8I). This is in contrast to *Aspergillus niger* complex, which had thick-walled, smooth, and hyaline stipes, and large, round vesicles with biseriate phialides and metulae over the entire surface (Figure 8A). Of note, both conidia were round with a relatively large diameter of 3–10 μm, black in *Aspergillus niger* complex and light yellow in *Aspergillus flavus* complex (Figures 8A,I). In this case, the combination of colony and microscopic morphology can be more effective in distinguishing *Aspergillus flavus* complex from *Aspergillus niger* complex.

Second, compared with the misidentified results of human readers (*n* = 12), only one image of *Aspergillus flavus* complex was misidentified as *Aspergillus fumigatus* complex in the initial model test set, but as a not-credible identification (Figure 8D), which indicates that the ability of human readers to distinguish *Aspergillus flavus* and *Aspergillus fumigatus* complexes needs to be improved by fungal morphologic training.

Third, *Aspergillus fumigatus* complex and *Aspergillus terreus* complex were also difficult to distinguish using morphology by XMVision Fungus AI (*n* = 2, manually collected test sets) and human readers (*n* = 9). The microscopic morphology of an overmature *Aspergillus fumigatus* complex isolate (cultured for 7 days in Figure 8F) was similar to that of *Aspergillus terreus* complex (Figure 8E), with both phialides on the upper two-thirds and similar long chains of conidia. Some obvious differences are that the conidiophore of *Aspergillus fumigatus* complex present pear-shaped vesicles, short stipes, and absent metulae with uniseriate phialides (Figure 8C), whereas *Aspergillus terreus* complex have vesiculate conidiophores with characteristic fan-shaped heads, which present smooth and hyaline stipes and dome-shaped vesicles with long, cylindrical metulae and biseriate phialides; besides, *Aspergillus terreus* complex may show chlamydospores (Figure 8E).

Fourth, additional images of *Aspergillus versicolor*/*Aspergillus sydowii* in initial model test set (*n* = 12) were not distinguished from *Aspergillus nidulans* by XMVision Fungus AI. It should be noted that *Aspergillus nidulans* presented distinct foot cells branching at 90° angles (Figure 8G). As for *Aspergillus versicolor*/*Aspergillus sydowii* (Figure 8H), they could not be distinguished from their microscopic appearances alone. Both conidiophores showed smooth and hyaline stipes, round vesicles with metulae, and biseriate phialides over the entire surface along with reduced *Penicillium*-like heads, which may be the reason they were misidentified as *Penicillium* spp. by human readers (*n* = 7).

Finally, a few images of *Syncephalastrum racemosum* from the manually collected test sets were misidentified as *Aspergillus flavus* complex by XMVision Fungus AI (*n* = 1) and human readers (*n* = 3). As an example of *Syncephalastrum racemosum* (Figure 8J), the broad hypha without septa, short stipes with branches, and finger-shaped tubular sporangia on the vesicle containing spores in chains were observed under the microscope, and they needed to be carefully distinguished from *Aspergillus flavus* complex (Figure 8I).

## Discussion

Multiple methods are used to identify moulds cultured from clinical samples, including morphologic identification, MALDI-TOF MS, and molecular sequencing methods. In our study, we developed XMVision Fungus AI using deep learning techniques, and nine taxa of clinical common moulds were successfully identified with an accuracy of 93.00% during the final testing.

Although the molecular sequencing method is regarded as the golden standard of fungal identification, because of the high cost of testing, complicated specimen pre-processing, and lacking of test standardization, it has been difficult to promote its use in clinical laboratories. Currently, the molecular sequencing method is more often used to identify the isolates with unusual phenotypic profiles and rare species (Balajee et al., 2007, 2009).

Morphologic identification methods, such as traditional culture methods and direct microscopic examination, have been always played a dominant role in mould identification. The conventional culture method is recognized as a golden standard for pathogen detection, and the positive rate of pathogen detection can be improved by clinical specimens cultured under certain conditions (Lefler et al., 1981). Direct microscopic examination is used to directly observe fungal morphologic characteristics in clinical specimens stained with 10% KOH, Gram, or lactophenol cotton blue (Kaliamurthy et al., 2011; Lin et al., 2021). However, it is difficult to observe the complete and typical fungal morphology in clinical specimens, which does not lead to accurate fungal species identification. Considering *Aspergillus* spp. as an example, when dichotomous acute angle branching septate hyphae or *Aspergillus* conidia are found in clinical specimens, a diagnosis of *Aspergillus* species infection can be provided (Ledoux et al., 2020), but the specific species cannot be determined.

Morphologic identification requires microbiological professionals to distinguish phenotypic traits such as colony structure (Li et al., 2017), colour, and growth rate as well as microscopic characteristics such as the structure of conidiophore and the size of conidia. Otherwise, misidentification is likely and the accurate identification of species is difficult. However, professionals in fungal morphologic identification are still lacking in China.

Our study also confirmed that different levels of mould identification experience in human readers lead to different performance levels, and their abilities could be raised by training (Figure 6). Compared with the direct image identification performed human readers, XMVision Fungus AI obtained a higher overall accuracy of 92.31%, with no significant difference in mould identification ability with respect to professional microbiologists (Figure 6). Notably, XMVision Fungus AI currently has only achieved such identification performance on microscopic images and has not been trained to identifying colony characteristics. Although microbiological professionals often combine colony characteristics with microscopic characteristics to identify and diagnose clinical specimens, we note that some taxa are difficult to identify using only phenotypic traits because of their similar colony appearances. For example, the colony appearances should be distinguished in *Aspergillus fumigatus*, *Aspergillus nidulans*, *Aspergillus versicolor*, and *Penicillium* spp. (Campbell et al., 2013). Of course, training XMVision Fungus AI to identify colony morphology of moulds is considered in our next plan.

During the last decades, MALDI-TOF MS has been widely used in the identification of bacteria and yeasts in clinical laboratories, because of its relatively rapid and accurate identification, convenient operation and a high-cost performance (Schubert and Kostrzewa, 2017; Peiffer-Smadja et al., 2020). However, MALDI-TOF MS has not been extensively used for mould identification because of the complex phylogenetic relationships between species and even more complicated morphology, which can make it difficult to extract fungal proteins (Peng et al., 2019), as well as insufficient fungal coverage in the database (Li et al., 2017). Except for *Syncephalastrum racemosum* and *Penicillium oxalicum*, which were not available in the VITEK^®^ MS V3.2 system’s database, the other isolates in our study could be correctly identified to the complex or species level. Of course, we believe that other MALDI-TOF MS database can cover thousands of fungal species, possibly including *Syncephalastrum racemosum* and *Penicillium oxalicum*. However, VITEK^®^ MS V3.2 system was only available in our study to identify these strains of the evaluation set and just to be compared with XMVision Fungus AI. However, *Aspergillus lentulus* could be distinguished from *Aspergillus fumigatus* by VITEK^®^ MS, which would help avoid inappropriate therapy because of its potential resistance to multiple antifungals (Arastehfar et al., 2019). In addition, because of the expensive equipment required, MALDI-TOF MS is not generally affordable for all clinical laboratories in China, especially those in primary hospitals.

Although XMVision Fungus AI is also based on the traditional fungal culture method, it can identify the strains to genus or complex level in less time than direct microscopic examination or MALDI-TOF MS. In addition, XMVision Fungus AI can achieve lower cost and more convenient operation, and hence it is more suitable for primary hospitals in China to assist in the identification of clinical moulds. Because XMVision Fungus AI identification only requires fungal microscopic images, primary hospitals only need to provide standard images for remote consultation. In comparison, clinical specimens are required for MALDI-TOF MS identification, and a remote consultation platform for MALDI-TOF MS may be more difficult to be realized than one for XMVision Fungus AI.

However, XMVision Fungus AI is still in the early stage of development, and there are still many problems to be solved. First, although XMVision Fungus AI is not trained to identify some *Aspergillus* subspecies, XMVision Fungus AI is also unable to achieve the distinction between the subspecies because they share the same or similar microscopic structural features, unless significant microscopic differences existed between subspecies. Therefore, *Aspergillus niger* and *Aspergillus tubingensis*, as well as *Aspergillus fumigatus* and *Aspergillus lentulus*, can only be identified the at the complex level by XMVision Fungus AI (Table 2). However, different subspecies may have different pathogenicity and drug susceptibility profiles, which may prevent effective clinical therapy (Li et al., 2017); however, this is always a drawback of morphologic identification. In addition, the subspecies of *Penicillium* spp. and *Fusarium* spp. were only identified at each genus level (Table 2). Although molecular sequencing methods such as polymerase chain reaction and real-time polymerase chain reaction were useful for fungal identification, no discrimination could previously be made between *Aspergillus versicolor* and *Aspergillus sydowii* (Libert et al., 2017). Moreover, the sporangiophore and merosporangia of *Syncephalastrum* spp. could also be mistaken for *Aspergillus* spp. if the isolate was not examined carefully (Kidd et al., 2016).

Second, due to the limitation of mould image acquisition conditions, the training images did not cover all features of each taxon, such as the immature, mature and aging stages, or some subspecies. In this case, the standard operation procedure (SOP), such as fungal culture periods, staining methods, matching microscopic settings, and image acquisition requirements, of XMVision Fungus AI should be strictly followed to achieve accurate identification. In our study, a similar morphology of *Aspergillus flavus* complex and *Aspergillus niger* complex was found during their juvenile stages (cultured for 2 days; Figure 8B), because the requirements of the SOP for a culture period of 3–5 days was not met for these mould taxa. Likewise, the overmature *Aspergillus fumigatus* complex (cultured for 7 days) was similar to the morphology of *Aspergillus terreus* complex (Figure 8F), which again did not satisfy the required 3–5 days for culture. In addition, the accuracy of 68.46% on test set B was significantly lower than that of 93.00% on test set A (Figure 5). This was because the mould images were acquired from the different settings of the five hospitals, who might not have strictly followed the SOP to perform the evaluation of XMVision Fungus AI. To enhance the ability of mould identification by XMVision Fungus AI, ACE processing was found in our study to effectively improve the quality of images captured from different settings or without a standard (*p* < 0.0001) through normalization of the background colour bias and colour equalization in the different areas of the raw images.

Additionally, the overfitting phenomenon occurred in our study, leading to a model with higher accuracy when identifying the initial model test set with the same origin as the training set. One reason for the overfitting was the performance difference in the image acquisition settings. Another reason was that the training sample batch was coupled with the identification, and the model learned features that were irrelevant to the actual identification, such as the background colour and brightness. ACE processing is a way to alleviate the overfitting by normalizing the images from different settings and batches.

A previous study demonstrated the potent broad-spectrum ability of the Xception convolutional neural network model to detect *Aspergillus* spp. strains based on the digital images of colonies (Ma et al., 2021). To the best of our knowledge, no attempt has been made to specifically identify moulds based on their microscopic characteristics. In contrast to other studies (Ren et al., 2017; Aradhya et al., 2021; Su et al., 2021; Zhang et al., 2021), we adopted the ResNet-50 model for end-to-end learning without structural labels, which was conductive to the development of cross-field research and development work to solve the data labelling problem. Additionally, if a model suitable for sample complexity is selected, excellent results can be produced by training with a large sample. However, the ResNet-50 model is not suitable for training with a small sample, and this requires a large workload for training preparation (Yu et al., 2021). Moreover, the number of data in the training set for identification needs to be balanced and the image quality needs to be consistent; otherwise, the overfitting phenomenon easily occurs. However, the transfer learning capability of the ResNet-50 model is somewhat weak.

XMVision Fungus AI is an assistive approach for the identification of clinical common moulds, which has a high commercial application prospect and is more suitable for primary hospitals in China or other developing countries. To further extend the application of XMVision Fungus AI to identify pathogenic moulds in practice, more microscopic images of clinical strains will be collected from other hospitals (more different acquisition settings) and used to build the national database of fungal images covering more taxa, different growth stages, different staining methods, and a wide range of acquisition conditions. In future, we will attempt to move the implementation of XMVision Fungus AI from computers to mobile devices to achieve more convenient and intelligent point-of-care testing of mould identification.

## Data availability statement

The raw data supporting the conclusions of this article will be made available by the authors, without undue reservation.

## Author contributions

RJ wrote the article. RJ, X-LX, JL, GZ, and W-HY carried out most work on XMVision Fungus AI model training. Y-CX and X-LY proposed and designed XMVision Fungus AI model. H-QL provided the technical guidance and support. Y-CX and T-SS provided critical review on the article. All authors contributed to the article and approved the submitted version.

## Funding

This work was supported by the National Major Science and Technology Project for the Control and Prevention of Major Infectious Diseases of China (2018ZX10712001); Beijing Key Clinical Specialty for Laboratory Medicine-Excellent Project (No. ZK201000); the National Key Research and Development Program of China (2021YFC2302005); the Chinese Academy of Medical Sciences (CAMS) Innovation Fund for Medical Sciences (2021-I2M-1-044 and 2021-I2M-1-038); Special Foundation for National Science and Technology Basic Research Program of China (2019FY101200); the National Nature Science Foundation of China (81971979, 81572057, and 81801989).

## Conflict of interest

X-LY and H-QL were employed by companies Beijing Hao Chen Xing Yue Technology Co., Ltd., China and Beijing Xiaoying Technology Co., Ltd., China, respectively. W-HY, Y-CX, RJ, GZ, X-LX, and X-LY are co-patentee of an invention patent named as ‘An invention related to a morphologic identification method of moulds, devices, and medium’, which was granted on Jan 14th 2022 with a patent number: ZL 2020 11523426.7.

The remaining authors declare that the research was conducted in the absence of any commercial or financial relationships that could be construed as a potential conflict of interest.

## Publisher’s note

All claims expressed in this article are solely those of the authors and do not necessarily represent those of their affiliated organizations, or those of the publisher, the editors and the reviewers. Any product that may be evaluated in this article, or claim that may be made by its manufacturer, is not guaranteed or endorsed by the publisher.

## Footnotes

^1^ https://www.blast.ncbi.nlm.nih.gov/Blast.cgi
