# Supplementary material for: Morphologic identification of clinically encountered moulds using a residual neural network
Source: Front Microbiol. 2022 Oct 14;13:1021236. doi: 10.3389/fmicb.2022.1021236 (PMC9614265; doi:10.3389/fmicb.2022.1021236)
Supplement: Supplementary file 1 [file Data_Sheet_1.PDF]

TABLE S1 A list of initial fungal resource

**Taxa**	**No. of isolates**
Aspergillus niger complex	73
Fusarium spp.	37
Aspergillus fumigatus complex	37
Penicillium spp.	33
Aspergillus flavus complex	32
Aspergillus terreus complex	29
Aspergillus nidulans	25
Aspergillus sydowii/Aspergillus versicolor	16
Syncephalastrum racemosum	13
Rhizopus spp.	9
Geotrichum spp.	9
Trichoderma spp.	8
Mucor spp.	8
Talaromyces spp.	7
Exophiala dermatitidis	6
Trichophyton spp.	6
Sporothrix schenckii	5
Lichtheimia spp.	5
Scedosporium spp.	5
Aspergillus ustus	4
Microsporum spp.	4
Cladophialophora spp.	2
Aspergillus lentulus	2
Cunninghamella spp.	2
Bipolaris spp.	2
Aspergillus tamarii	2
Acremonium spp.	2
Alternaria spp.	2
Scopulariopsis spp.	2
Beauveria spp.	2
Aspergillus ochraceus	1
Monascus ruber	1
Curvularia lunata	1
Neurosporacrasa	1
Eutypella spp.	1
Aspergillus pseudoglaucus	1
Aspergillus clavatus	1
Phoma spp.	1
Paecilomyces spp.	1

TABLE S2 Clinical information of strains used for an evaluation set

Strain number	ID number	Species	Isolation sites	Clincial diagnosis
No.1	19AA0474	*Syncephalastrum racemosum*	Skin swab	Dermatocyst
No.2	19AA0779	*Aspergillus nidulans*	Sputum	Bronchitis
No.3	19AA0337	*Aspergillus niger*	Ear swab	Otomycosis
No.4	19AA0746	*Aspergillus tubingensis*	Sputum	PA
No.5	19Z10672	*Aspergillus flavus*	Tracheal aspirate	IPA
No.6	19AA0520	*Fusarium verticillioides*	Eye swab	Keratitis
No.7	19AA0628	*Fusarium solani*	Blood	Leucocythemia
No.8	19AA0462	*Penicillium oxalicum*	Blood	Fever
No.9	19AA0521	*Penicillium citrinum*	Ear swab	Otomycosis
No.10	19AA0550	*Aspergillus terreus*	Ear swab	Otomycosis
No.11	16R8468	*Aspergillus lentulus*	BALF	IPA
No.12	19AA0679	*Aspergillus fumigatus*	BALF	IPA
No.13	19AA0179 20z09626	*Aspergillus sydowi/Aspergillus versicolor*	Sputum	Bronchitis Pneumonia

Abbreviation: PA, pulmonary aspergillosis; IPA, invasive pulmonary aspergillosis; BALF, bronchoalveolar lavage fluid

TABLE S3a-S3c Comparison of commonly misidentified mould taxa between XMVision Fungus AI and Human readers

3a. Identification results from initial model test Set

Species	*A. flavus* complex	*A. fumigatus* complex	*A. nidulans* complex	*A. niger* complex	*A. terreus* complex	*A. versicolor/ A. sydowi*	*Fusarium* spp.	*Penicillium* spp.	*S. racemosum*	Total
***A. flavus* complex**	56	1		2						59
***A. fumigatus* complex**		73								73
***A. nidulans* complex**			102							102
***A. niger* complex**				182				1		183
***A. terreus* complex**					99					99
A. versicolor/A. sydowi			12			153				165
***Fusarium* spp.**							95			95
***Penicillium* spp.**								158		158
S. racemosum									93	93
Total	56	74	114	184	99	153	95	159	93	1027

3b. Identification results from manually collected test sets (test Set A)

Species	*A. flavus* complex	*A. fumigatus* complex	*A. nidulans* complex	*A. niger* complex	*A. terreus* complex	*A. versicolor/ A. sydowi*	*Fusarium* spp.	*Penicillium* spp.	*S. racemosum*	Total
***A. flavus* complex**	22			9						31
***A. fumigatus* complex**		27			2					29
***A. nidulans* complex**			46	1						47
***A. niger* complex**				41						41
***A. terreus* complex**				2	36					38
A. versicolor/A. sydowi						3				3
***Fusarium* spp.**				1			59	1		61
***Penicillium* spp.**			1		3			29		33
S. racemosum	1								16	17
Total	23	27	47	54	41	3	59	30	16	300

3c. Identification results from Human readers

Species	*A. flavus* complex	*A. fumigatus* complex	*A. nidulans* complex	*A. niger* complex	*A. terreus* complex	*A. versicolor/ A. sydowi*	*Fusarium* spp.	*Penicillium* spp.	*S. racemosum*	Others	Total
***A. flavus* complex**	23	12		4	1					5	45
***A. fumigatus* complex**	8	61	1		9	1			1	9	90
***A. nidulans* complex**		1	22		1	6		5		10	45
***A. niger* complex**	12			73						5	90
***A. terreus* complex**	1	2	3		24	3		4		8	45
A. versicolor/A. sydowi			1		1	27		7		9	45
***Fusarium* spp.**							77			13	90
***Penicillium* spp.**			8			1	2	71		8	90
S. racemosum	3								29	13	45
Total	47	76	35	77	36	38	79	87	30	80	585
